# Outcomes of infants undergoing surgery for oesophageal atresia and trachea-oesophageal fistula at a tertiary referral hospital in Tanzania

**DOI:** 10.1371/journal.pgph.0005851

**Published:** 2026-09-10

**Authors:** Masawa Klint Nyamuryekung’e, Kasusu Klint Nyamuryekung’e, Rajabu Athumani Bakari, Godfrey Sama Philipo, Petronilla Ngiloi

**Affiliations:** 1 Department of Surgery, The Aga Khan University East Africa Medical College, Dar es Salaam, Tanzania; 2 Community Department, Muhimbili University of Health and Allied Sciences, Dar es Salaam, Tanzania; 3 Department of Surgery, Muhimbili National Hospital, Dar es Salaam, Tanzania; 4 College of Surgeons East, Central, and Southern Africa, Arusha, Tanzania; The University of British Columbia, CANADA

## Abstract

Oesophageal atresia (OA/TOF) is a neonatal surgical emergency with high mortality in low- and middle-income countries, where delayed diagnosis, pneumonia, and limited perioperative capacity contribute to poor outcomes. Contemporary data from Tanzania are scarce. This study assessed preoperative delays, in-hospital preoperative mortality, 14-day postoperative mortality, and associated factors among neonates with OA/TOF at a national referral hospital. A 10-year retrospective review (2012–2022) was conducted at Muhimbili National Hospital for neonates with OA/TOF. Descriptive statistics and bivariate associations with preoperative and postoperative mortality were done. Exact one-sample binomial tests compared observed 14-day survival in Waterston classes B and C with historical expected survival. Time-fixed binary logistic regression identified independent predictors of 14-day postoperative mortality. Preoperative mortality was associated with lower gestational age (p = 0.010), greater pneumonia severity (p = 0.009), and fewer completed investigations for associated anomalies (p = 0.027). Fourteen-day postoperative mortality was 82.7% (43/52). Survival in Waterston class B (18.2%) was significantly lower than expected (p < 0.001), while class C survival (15.8%) was significantly higher than expected (p < 0.001). Median postoperative survival was 6 days (95% CI 2.88–7.11). Survival did not differ by Waterston class, pneumonia severity, or birth-to-surgery timing. Surgery within the first week was associated with lower odds of 14-day mortality (aOR 0.34, 95% CI 0.12–0.97). OA/TOF outcomes in this setting are marked by substantial preoperative and postoperative mortality. This study adds new insight by quantifying where delays occur and showing that most mortality accumulates before and shortly after surgery, indicating system-level constraints similar to those reported across sub-Saharan Africa. Early surgery within the first week was a predictor of improved survival. These findings provide locally generated evidence to strengthen neonatal surgical and perioperative capacity.

## Introduction

Birth defects (BD) are among the top five causes of mortality in under-fives globally, affecting 6% of all births worldwide annually, with an associated mortality of 3.3 million children. Due to higher birth rates and limitations in health systems capacity in low-middle-income countries (LMIC), 94% of BDs and the associated 95% of mortality occur in this region [1]. The 63^rd^ World Health Resolution on birth defects recommends BD control strategies that include strengthening early detection and referral capacity, raising BD awareness, research and collaboration and improving the BD tertiary-level care and control capacity [1]. The existing capacity, and the status of implementation of these strategies is largely unknown in many health systems in LMIC and are implicated as the major driver of limited positive outcomes.

Oesophageal atresia (OA) with tracheoesophageal fistula (TOF) occurs at an incidence of 1 in 3,000–5,000 live births worldwide [2]. In high-income countries, advances in neonatal intensive care and surgical techniques have driven postoperative survival above 90%, with operative mortality now under 10% [3–5]. By contrast, low- and middle-income countries—particularly in sub-Saharan Africa—report postoperative mortality rates of 30%–90%, largely due to delayed diagnosis, aspiration pneumonia, and sepsis stemming from limited referral pathways and scarce perioperative resources [4,6]. In Tanzania, case reports highlight late referrals and high sepsis-related mortality, indicating the need for larger cohort studies at tertiary centres to assess management, challenges, and outcomes [7].

Oesophageal atresia and tracheoesophageal fistula (OA/TOF) is a critical, non-visible, gastrointestinal BD, amenable to emergent surgical intervention early after birth, requiring postoperative critical care and mechanical ventilation to prevent mortality [8,9]. Higher birth rates in LMICs potentially imply higher number of cases of OA/TOF in this region. Apart from the paediatric surgical expertise required; the associated anomalies, intrauterine growth restriction, low birth weight (LBW), and prematurity of the newborns necessitate a robust health system capacity for optimal outcomes. This spans the entire range of interventions from early detection to neonatal surgical capacity at tertiary-level care health facilities. The outcomes of OA/TOF therefore reflect the health systems’ capacity for managing a complex urgent surgical neonate [10,11].

One of the main factors driving excess mortality in resource-limited health systems is an overwhelmed and largely unavailable necessary neonatal surgical capacity causing management delays [12]. Pre-hospital delays due to limitations in systems necessary for early BD detection, absence of appropriate referral pathways, in-hospital pre-operative delays due to limited and overwhelmed neonatal intensive care and surgical capacity, lack of timely evaluation and optimization of the surgical neonate, all contribute to a worsening of the neonate’s physiological reserve during the surgical waiting period [13]. Birth weight (BWT) which is an indirect marker of the fragility of the neonate, aspiration pneumonia, and sepsis remain important predictors of OA/TOF mortality in Sub-Saharan Africa [6,14,15]. It is suspected that significant, yet unaccounted preoperative mortalities occur during these delays. For those neonates that survive to surgical intervention, systems’ limitations increase the risk of adverse postoperative outcomes [14,16].

In Waterston’s (1962) OA/TOF prognostic classification [14], neonates considered to be relatively well (Class A) have an expected post-operative survival is 95%. In class C group, those with either severe pneumonia, severe associated anomaly, or weight less than 1800 gm at birth the expected survival is 6%. For those falling between these two classes, Class B, the expected survival was 79% [17]. These outcomes are expected if neonates reach a tertiary-level facility with adequate resources and experience, permitting surgical intervention for Class A neonates within 24–48 hours of presentation. Currently, in well-resourced health systems, the only persistent prognostic factor is duct-dependent congenital heart disease, with survival rates of more than 95% [18,19].

This study reviewed 10-year in-hospital outcomes for OA/TOF at a single tertiary hospital in Tanzania. During the study period, this facility was one of only two in the country with paediatric surgeons operating on neonates with this condition. The adequacy of neonatal anaesthesia, neonatal intensive care, and neonatal surgical capacity in these facilities had not been previously determined. We traced the journeys of neonates with OA/TOF from birth to within 14 days post operatively. We aimed to determine management delays, in-hospital pre-operative, and 14-day post operative mortality and describe the associated factors. The findings of this study can be used to determine the current tertiary-level care capacity for managing OA/TOF. Furthermore, describing the associated factors will unveil possible opportunities for improving the status.

## Methodology

### Ethics Statement

This study received ethical approval from the Muhimbili University of Health and Allied Sciences Research Ethics Committee (MUHAS-REC), reference MUHAS-REC-01-2023-1513, and permission to access clinical records from the Muhimbili National Hospital Institutional Review Board, reference MNH/TRCU/IRB/Perm/2023/256. The study involved retrospective review of existing medical records, and the requirement for informed consent was waived by both committees. All data were fully anonymized prior to analysis, and no identifiable patient information was collected. Data extraction was conducted between 01 May 2023 and 01 October 2023. All procedures were performed in accordance with institutional guidelines, national regulations, and the principles of the Declaration of Helsinki.

### Study site and period

A 10-year retrospective chart review was conducted between January 2012 to December 2022 at Muhimbili National Hospital.

### Participants

All neonates with radiologically confirmed OA/TOF were included. Admission records were reviewed and identified by ICD-9 code that the study site was using. Charts were then reviewed, and diagnosis was confirmed based on the radiological criteria were a coiled NGT in the proximal oesophageal pouch, with or without the presence of gas in the GIT distally. Those who could not be determined to have either survived or died from admission to within 14 days postoperative were excluded because of missing data. All neonates meeting the inclusion criteria were included. Cases with missing outcome data were excluded from analysis using listwise deletion.

### Variables

Neonatal baseline characteristics included the presence and severity of pneumonia at presentation, the number of days from birth to diagnosis, from diagnosis to operative intervention, and from birth to operative intervention. Pneumonia severity was classified using chest x ray findings, as the original Waterston study [17], and the need for supplemental oxygen as follows: Class A—no radiographic infiltrates and no supplemental oxygen requirement; Class B—radiographic infiltrates without oxygen requirement; Class C—radiographic infiltrates with supplemental oxygen requirement to maintain SpO_2_ > 95%. Birth weight was captured in grams and transformed to birth weight category as per WHO; Normal Birth weight (>2,500grms), low birth weight (1500–2,500 grams), very low birth weight (1000 to <1500 grams), and extremely low birth weight (<1000 grams). Gestational age at birth was captured in weeks transformed into gestational age at birth category as per WHO into, term (>37 weeks) moderate to late preterm (32 to <37 weeks), very preterm (28 to <32 week), and extremely preterm (< 28 weeks). Birth to diagnosis time was captured in days and transformed into birth to diagnosis time category; within 1 day, within 2–4 days, within 4–7 days, beyond 1 week. The time from birth to surgery was captured in days, and also categorized into three groups, those operated within the first week (7 days), within the second week, and beyond the second week of birth. Assessment of associated anomalies was done using ECHO, thoraco-abdominal radiograph, and kidney-ureter-bladder ultrasound. At the study site, investigations for associated anomalies are performed selectively and only when clinically indicated. Therefore, infants who were not evaluated (e.g., no echocardiogram, abdominal X‑ray, or KUB ultrasound) were considered clinically normal at the time of care, and were captured as such in this study. The associated anomalies and their severity were captured according to Waterston’s classification, mild/none, moderate, and severe, based on the resulting physiological derangement.

All neonates were admitted to the neonatal intensive care unit (NICU). During the study period, total parenteral nutrition (TPN) was intermittently available and therefore not routinely provided. Oral secretions were managed using head‑up positioning and intermittent oropharyngeal suctioning, as continuous upper pouch suction was not available. Preoperative optimization was undertaken jointly by neonatology, pediatric surgical, and anesthesia teams. RAB extracted data. A 10% sample was double-checked for accuracy by a second reviewer, MKN.

Preoperatively, the neonates were grouped into Waterston categories, A, B and C. Category A included the lowest-risk infants, defined as those with a birth weight over 2.5 kg who are otherwise healthy, lacking pneumonia or other major congenital anomalies. Category B included an intermediate-risk category, consisting of infants with a birth weight between 1.8 kg and 2.5 kg, or higher-weight infants who present with moderate pneumonia and/or moderate congenital anomalies. Finally, Category C comprised the highest-risk infants, defined either by a very low birth weight under 1.8 kg or by the presence of severe pneumonia or severe congenital anomalies, regardless of birth weight.

**Fig 1:**
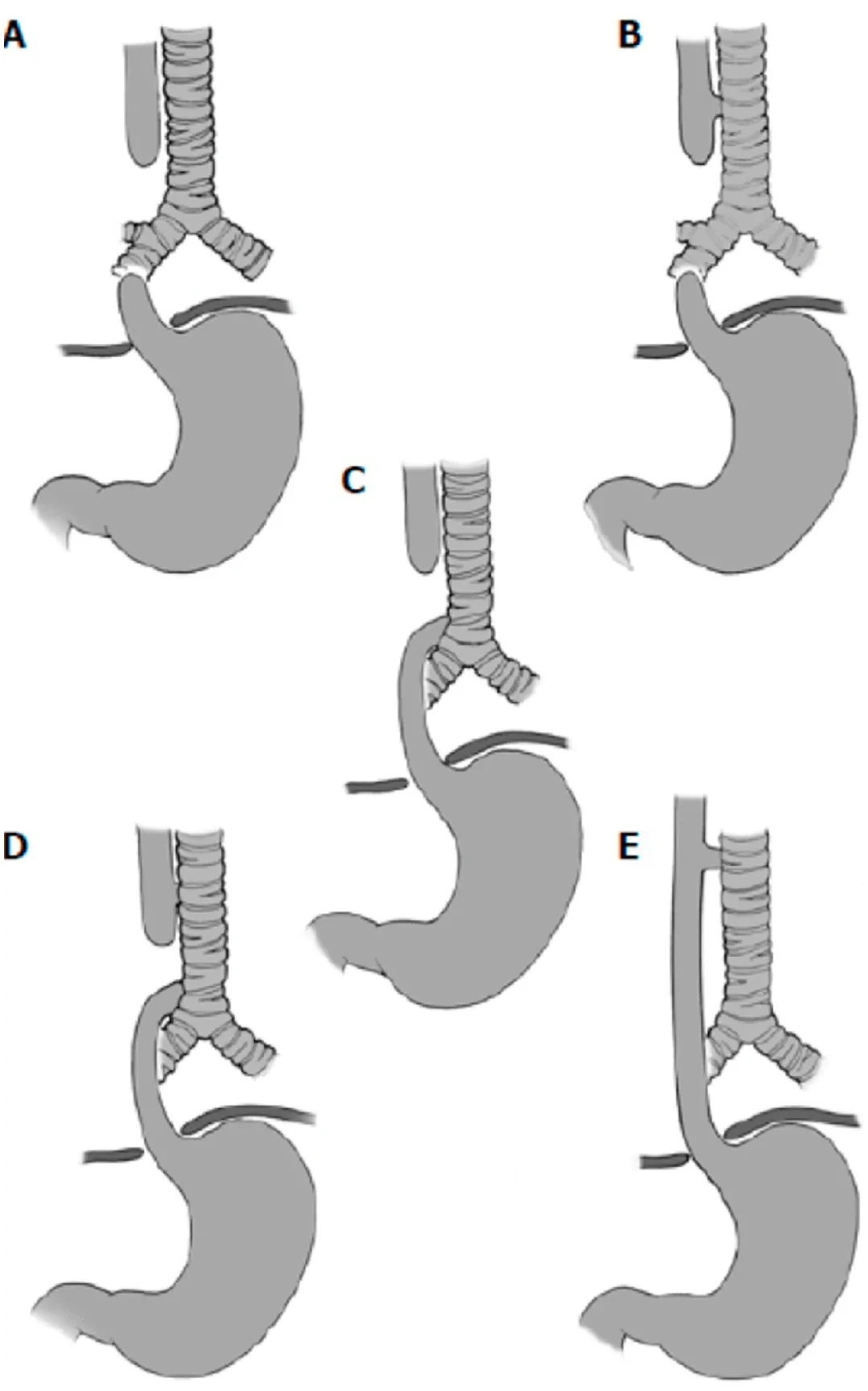
Gross anatomical classification of oesophageal atresia with or without tracheoesophageal fistula (OA/TOF) [20]. Schematic representation of the major OA/TOF subtypes **(A–E)**, illustrating variations in esophageal discontinuity and tracheoesophageal fistula configuration.

In-hospital pre-operative mortality was defined as death from any cause occurring between hospital admission and the initial surgical intervention. In-hospital post-operative mortality was defined as death from any cause occurring in the hospital within 14 days of the initial surgical intervention. The surgical approach for all the neonates was left 4^th^ intercostal space, muscle-sparing thoracotomy. Intraoperatively, OA/TOF was categorised anatomically using the Gross classification as shown in Fig 1, based solely on macroscopic intraoperative examination; trachea-bronchoscopy was not available. Furthermore, OA/TOF was classified as short or long gap, based on the ability of the surgeon to perform primary anastomosis. During the study period, postoperative management typically included mechanical ventilation for 24–48 hours, depending on respiratory status, if extubation was achieved, then initiation of trans-anastomotic feeds proceeded, on postoperative day 5 an esophagogram would be done, and if the anastomosis is intact, removal of the orogastric tube would be done and progress to oral feeds. TPN was used only when available.

### Analysis

Neonatal baseline characteristics were summarised using frequencies, means with standard deviations (SD), or medians with interquartile ranges (IQR Q1 to Q3), according to data distribution as tested by Shapiro- wilk test. The association between birth weight, gestational age, time from birth to diagnosis, severity of pneumonia, and the proportion that completed at least one baseline investigation with in-hospital preoperative mortality were assessed using a chi-square test, or Fisher’s exact units for categorical variables, and student’s t-test, or Mann-Whitney U test for continuous variables, depending on the counts within groups and the sample distribution respectively. The observed 14-day postoperative survival for Waterston’s Class A, B, and C was compared to the expected 95%, 79% and 6%, respectively, using the Exact one-sample binomial test.

Postoperative survival is analysed using Kaplan–Meier curves. The event of interest will be 14-day postoperative mortality, with right-censoring applied to neonates who survive beyond 14 days postoperatively. Bivariate associations between survival and Waterston classification, pneumonia severity, and birth-to-surgery time categories will be assessed using either the log-rank test or the Breslow (generalized Wilcoxon) test, depending on whether the proportional hazards assumption is met. The proportional hazards assumption will be evaluated using graphical inspection of log–minus–log survival plots. Median survival times and their 95% confidence intervals will be reported as descriptive summaries derived from the Kaplan–Meier estimator, which does not rely on proportional hazards. Because early postoperative mortality represents a clinically relevant binary endpoint, time-fixed binary logistic regression will be used to identify independent predictors of 14-day postoperative mortality. A p-value of <0.05 will be considered statistically significant for all analyses. SPSS version 26 was used for all statistical analyses. This study was reported in accordance with the Strengthening the Reporting of Observational Studies in Epidemiology (STROBE) guidelines. A completed STROBE checklist is provided in S1 File STROBE Checklist

## Results

**Fig 2:**
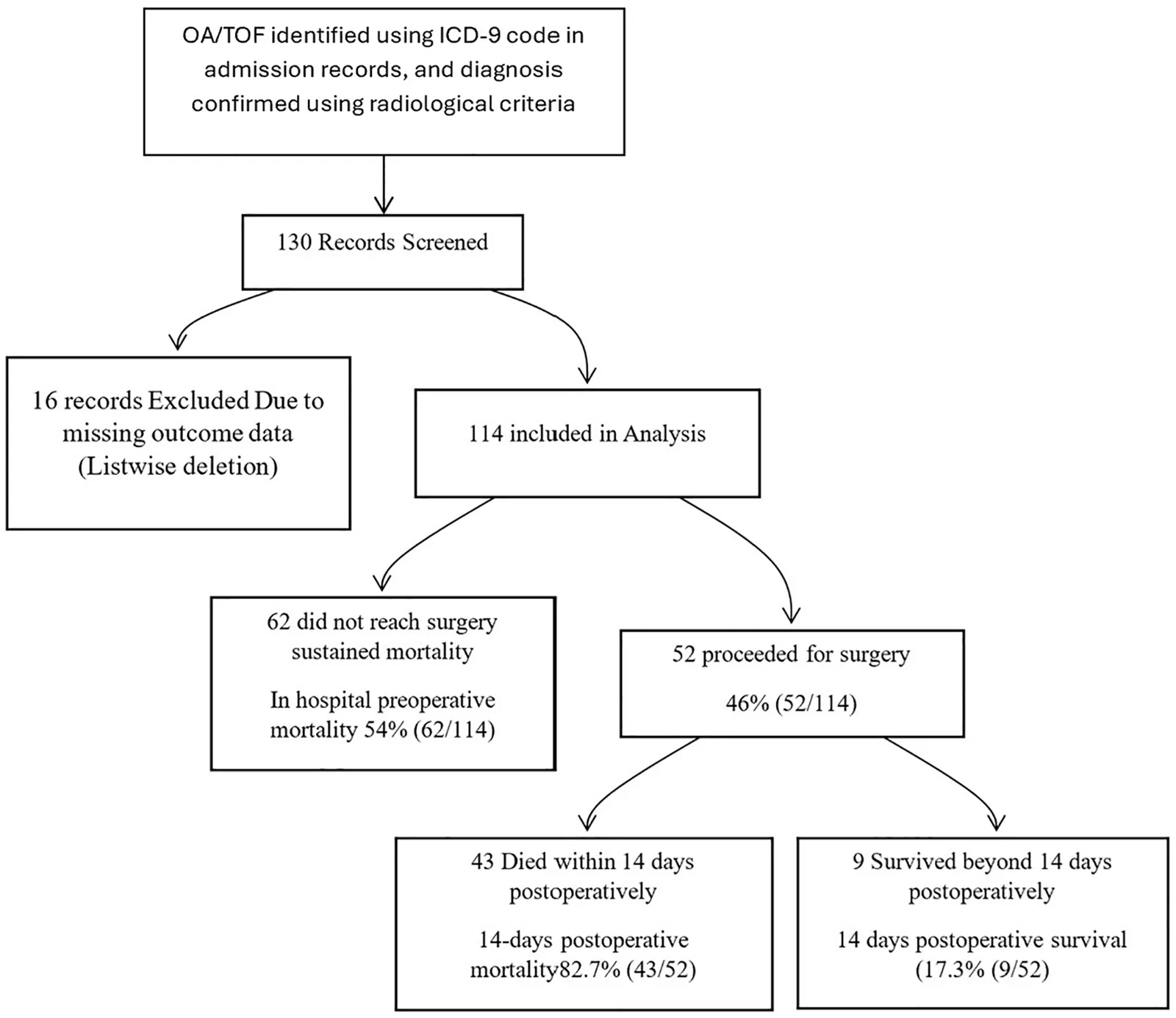
Flow of records screened, exclusions, and postoperative outcomes.

From the 130 neonates initially assessed, 114 had complete data and were included in the final study population. Sixteen neonates did not have a recorded survival status; these cases could not be classified as alive or deceased, and in several instances, the timing of death (pre‑ vs post‑operative) was unknown. Because survival is the primary outcome, listwise deletion was applied. Any imputation or sensitivity analysis would require unverifiable assumptions and risk introducing greater bias, see Table 1 on missing varaibles and participant flow shown in Fig 2.

**Table 1:** Showing missing variables.

Variable	Total (N)	Missing/Not done (n)	Percentage missing (%)	Notes/Reasons
**Preoperative variables**				
Sex	114	0	0%	
Gestational age	114	0	0%	
Birth weight	114	0	0%	
Referral Status	114	0	0%	
Chest X ray	114	0	0%	
Requirement for oxygen supplementation	114	0	0%	
Birth to diagnosis duration	114	0	0%	
ECHO investigation	114	34	30%	Considered normal
Abdominal pelvic X ray	114	108	95%	Considered normal
KUB USS	114	110	96%	Considered normal
Cranio-facial examination	114	0	0%	
Limbs examination	114	0	0%	
Anterior Abdominal wall	114	0	0%	
Chest wall	114	0	0%	
Perineum and anorectal area	114	0	0%	
External genitalia	114	0	0%	
				
Gestational age category	114	0	0%	
Birth Weight Category	114	0	0%	
Pneumonia Severity	114	0	0%	
Waterstone classification	114	0	0%	
**Intraoperative variables**				
Did the neonate undergo surgery	114	0	0%	
Reasons for not undergoing surgery	62	0	0%	
Diagnosis to undergoing surgery duration	54	0	0%	
Birth to undergoing surgery duration	52	0	0%	
Intraoperative findings	52	0	0%	
Surgical procedure done	52	0	0%	
**Outcome Variables**				
Was there in hospital mortality	130	16	12%	List-wise deletion
Was there pre preoperative mortality	114	0	0%	
Post-operative mortality	52	0	0%	
Day of postoperative mortality	52	0	0%	

Screening and inclusion pathway for 130 neonatal records, showing exclusions due to missing outcome data, preoperative mortality among those who did not reach surgery, and 14‑day postoperative survival and mortality among infants who underwent surgical repair.

Table 1 Summary of missing data across all pre-operative, intraoperative, and outcome variables. Selective investigations (e.g., echocardiography, abdominal X-ray, KUB ultrasound) were performed only when clinically indicated; infants not investigated were considered clinically normal. Missing in-hospital mortality data (12%) were handled using listwise deletion, as imputation of the primary outcome would have required unverifiable assumptions.

The vast majority (93%) were referred from lower-level health facilities. Gestational age at birth (Shapiro Wilks test-0.94, df- 114, p < 0.001), birth weight (Shapiro Wilks test-0.97, df- 114, p-0.004), birth to diagnosis (Shapiro Wilks test-0.87, df- 114, p < 0.001), diagnosis to surgery (Shapiro Wilks test- 0.85, df-52, P-0.001), and birth to surgery time (Shapiro Wilks test- 0.95, df-52, P-0.010) were not normally distributed. The included neonates had a median gestational age of 37 weeks (IQR 34.75 to 39 weeks) and a median birth weight of 2,500 grams (IQR 2090–3000 grams), with 50.9% (58/114) of the infants classified as preterm. Only 15.8% received a radiological diagnosis of oesophageal atresia/tracheoesophageal fistula (OA/TOF) within the first 24 hours of life, with a median interval from birth to radiological diagnosis of 5 days (IQR 3 to 9.25 day). Once the diagnosis was established, 35.1% (40/114) had no or only mild pneumonia, 42.1% (48/114) had moderate pneumonia, and 22.8% (26/114) had severe pneumonia.

During the preoperative period, none of the neonates underwent the full complement of recommended investigations for associated anomalies. All had a chest X-ray to confirm the diagnosis; 70.2% (80/114) had an echocardiogram; 5.3% (6/114) had an abdomino-pelvic X-ray; and 3.5% (4/114) had a kidney, ureter, and bladder ultrasound. Based on the documented physical examinations of all neonates, minor limb anomalies were present in 1.8% (2/114), and craniofacial anomalies in 2.6% (3/114), one being of anencephaly, a severe anomaly, and the other two cases of cleft lip and palate, moderate anomalies. Anorectal malformations were present in 4.4% (5/114), all of which are severe anomalies. Congenital heart diseases were present in 26% (30/114), with only one being a physiologically significant lesion, Tetralogy of Fallot, a severe associated anomaly. The others were acyanotic left-to-right shunts, mostly small atrial septal defects, ventricular septal defects, and patent ductus arteriosus, which were not hemodynamically significant, being mild anomalies. Vertebral anomalies were present in 2.6% (3/114), and urological anomalies in 0.9% (1/114), all of which were mild. According to Waterston’s prognostic classification, 56% (64/114) were class B, and 44% (50/114) C, with no neonates categorised as class A (Table 2).

**Table 2:** Baseline characteristics.

Characteristic	Value
Male-to-female ratio	1.3:1
Referred from lower-level hospitals	93% (106/114)
Gestational age median (Q1 to Q3)	37 weeks (IQR 34.75–39)
Preterm (<37 weeks)	51% (58/114)
Birth weight, median (Q1 to Q3)	2500 g (IQR 2090–3000)
Normal birth weight (≥2500 g)	45% (52/114)
Radiological diagnosis ≤24 h	15.8% (18/114)
Birth to diagnosis in days, median (Q1 to Q3)	5 days (IQR 3–9.25)
**Severity of pneumonia at presentation**	
None/mild	35.1% (40/114)
Moderate	42.1% (48/114)
Severe	22.8% (26/114)
**Investigations done**	
ECHO	70.2% (80/114)
Abdominal X-ray	5.3% (6/114)
KUB ultrasound	3.5% (4/114)
**Prevalence of determined anomalies**	
Congenital heart disease (CHD)	26% (30/114)
Vertebral anomalies	2.6% (3/114)
Urological anomalies	0.9% (1/114)
Minor limb anomalies	1.8% (2/114)
Craniofacial anomalies	2.6% (3/114)
Anorectal malformation	4.4% (5/114)
**Waterston classification**	
Class B	56% (64/114)
Class C	44% (50/114)

The table summarises demographic features, referral patterns, perinatal characteristics, timing of diagnosis and surgery, severity of pneumonia at presentation, investigations performed, prevalence of associated congenital anomalies, and distribution of Waterston risk classes in the study cohort (N = 114).

The in-hospital preoperative mortality rate was 54% (62/114) (95% CI 45.3%–63.5%). The most prevalent factors contributing to mortality included respiratory compromise with high ventilatory support requirements in 75% (47/62), predominantly due to pneumonia, persistent electrolyte imbalances in 30% (19/62), and ongoing haematological abnormalities in 26% (16/62) cases. There were no significant differences in birth weight (Mann–Whitney U = 1939.50; z = 1.87; two‑sided P = 0.062), time from birth to diagnosis (Mann–Whitney U = 1581.50; z = -1.74; two‑sided P = 0.861) or Waterston classification (χ² = 3.295, df = 2, P > 0.05) between those who survived and those who died before surgery. The survivors had a higher mean rank for gestational age (66.18 versus 50.22). The difference was statistically significant (Mann–Whitney U -2,063.50; z = -2.59; two‑sided P = 0.010). The standardised effect size (r) was 0.24, indicating a small to moderate effect. Similarly, the severity of pneumonia (χ² = 9.38, df = 2, P = 0.009), and the proportion that completed at least one investigation for associated anomalies in the preoperative period, 63% (39/62) for the non survivors compared to 82.7% (43/52) among those who survived to undergo operative intervention (χ2 = 7.24, df = 2, P = 0.027) were statistically significantly different in the two groups (Table 3).

**Table 3:** Showing comparisons between those who survived and sustained mortality in the preoperative period.

Characteristic	Survivors (n = 52)	Non-survivors (n = 62)	Statistic/ P-value
**Birth weight, grams** median (Q1 to Q3)	2700 (2200–3000)	2500 (I800 to 2913)	Mann–Whitney U = 1939.5; P = 0.063
**Gestational age, weeks** median(Q1 to Q3)	38.0 (36–39)	36.0 (34 [ ]–38)	Mann–Whitney U = 2063.5; **P = 0.010**
**Birth-to-diagnosis time, days** median (Q1 to Q3)	5 (3 to 8.75)	5 (2.7 to 10)	Mann–Whitney U = 1581.5; P = 0.861
**Severity of pneumonia at presentation**			**Chi-square = 9.38; df = 2; P = 0.009**
None/mild	15 (28.8%)	29 (46.8%)	
Moderate	28 (53.8%)	16 (25.8%)	
Severe	9 (17.3%)	17 (27.4%)	
**Completed ≥1 investigation for anomalies**	**43 (82.7%)**	**39 (63.0%)**	**Chi-square = 7.24; df = 1; P = 0.027**
**Waterston classification**			**Chi-square = 3.30; df = 1; P > 0.05**
Waterston B	33 (63.5%)	31 (50%)	
Waterston C	19 (36.5%)	31 (50%)	

Continuous variables were compared using the Mann–Whitney U test and categorical variables using the χ² test. Pneumonia severity was classified as none/mild, moderate, or severe based on clinical and radiographic findings. A *P*-value < 0.05 was considered statistically significant.

A total of 52 neonates underwent surgical intervention, representing 46% (52/114) of the cohort. These neonates had a median duration from diagnosis to operation of 7 days (IQR 4.25 to 10.75 days). The median birth to operation time was 14.0 days (IQR 9.0 to 18.75 days), distributed as follows; those operated within the first week of life 19% (10/52), within the second week of life 36.5% (19/52) and those operated beyond the second weeks of life 44% (23/52). Intraoperatively, OA/TOF was classified as follows; Gross type C, 92% (48/52) and type A, 8% (4/52), other Gross Types, B, D and E were not encountered in this cohort. Of those with type C, 85% (41/48) underwent primary repair, 5% (2/48) had fistula ligation only as part of a staged approach due to intraoperative instability, and 10% (5/48) underwent fistula ligation, gastrostomy, and oesophagostomy as their initial procedure due to long gap- inability to perform primary anastomosis intraoperatively. Those with gross type A, underwent gastrostomy, and oesophagostomy as their initial procedure.

**Fig 3:**
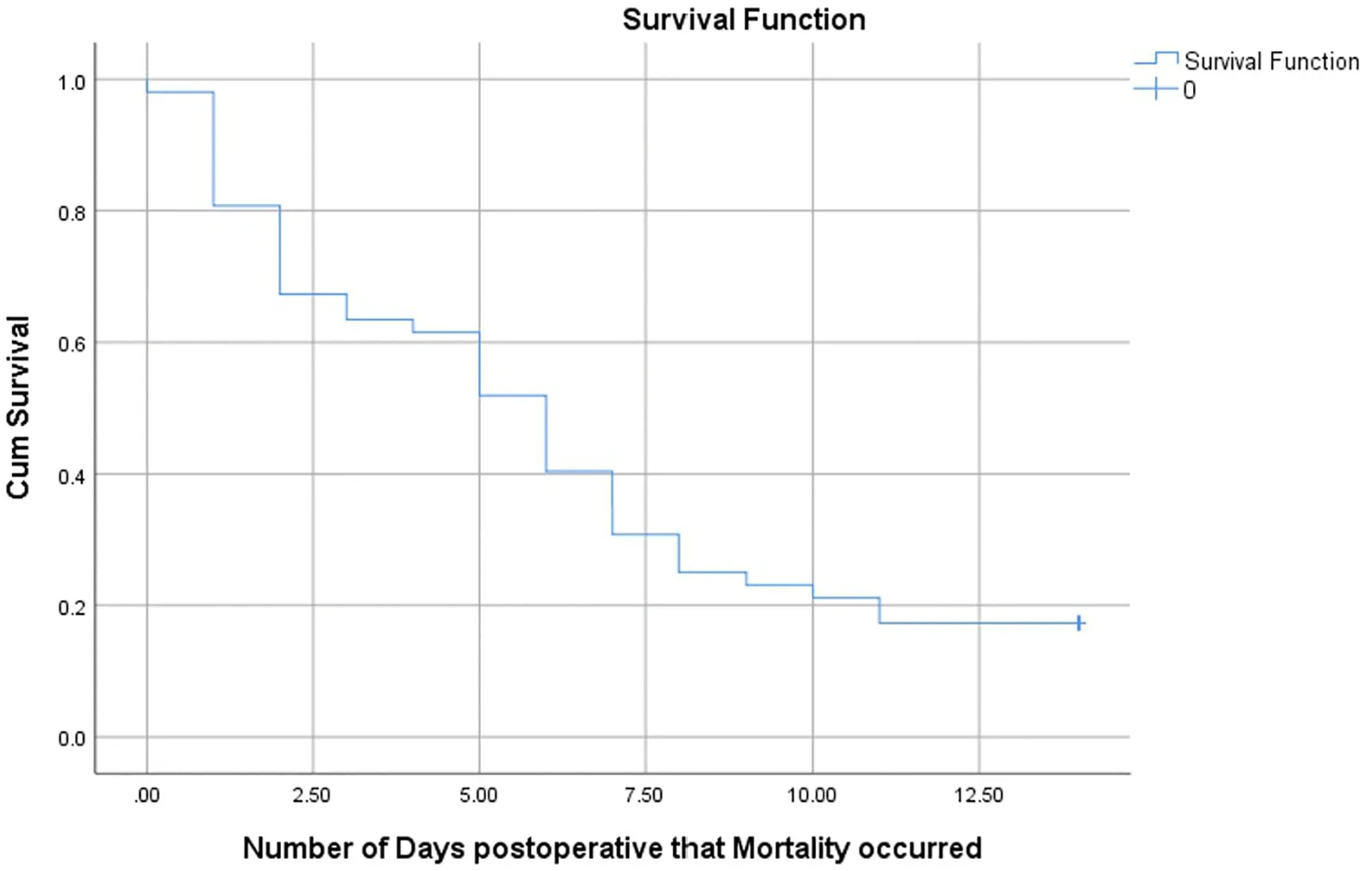
14-day Post operative survival. 14-day post operative survival curve for OA/TOF, 75% survival on day 2  ±± 0.483, 50% survival on day 6± ± 0.643 and 25% survival on day 8± ± 1.748. Median 14 day post operative survival of 6 days± 0.643.

Fourteen-day postoperative mortality was 82.7% (43/52) (Bernoulli SD 0.378; 95% CI [Wilson] 70.3%–90.6%). For neonates classified as Waterston class B, an exact one-sample binomial test was used to compare the observed 14-day outcome proportions to the expected 0.79 survival in the original Waterston study. In our cohort, survival for this group was 18.2% (6/33) (Clopper–Pearson 95% CI 7%–36%), significantly lower than expected (exact one-tailed p < 0.001). For those in Waterston C, the observed 14-day survival of 15.8% (3/19) (Clopper–Pearson 95% CI 3.6%–33.6%) was significantly higher than the 6% expected from the original Waterston study (exact one-tailed p < 0.001). Survival analysis revealed that 25% of deaths occurred by the second postoperative day, with a median survival time of six days (95% CI 2.88–7.11 days; Fig 3). Because the proportional hazards assumption was not satisfied and most events occurred early, Wilcoxon–Breslow tests were used. Survival comparisons between Waterston classes (χ² = 1.99, df = 1, p = 0.159), pneumonia severity (χ² = 1.174, df = 2, p = 0.556), and birth-to-surgery timing (χ² = 3.23, df = 2, p = 0.199) were nots tatistically significant (Table 4).

**Table 4:** Factors associated with 14‑day post operative survival.

Grouping	Category	14-day survival % (n/N)	Median survival (days) 95% CI	Statistical comparison	Test statistic	Test statistic
Overall survival	All operated neonates	17.3% (9/52)	6.0 (2.88–7.11)			
Waterston classification	B	18.2% (6/33)	6.0 (7–36)	Exact binomial vs expected 0.79	One-tailed p < 0.001	**<0.001**
Waterston classification	C	15.8% (3/19)	3.0 (3.6–33.6)	Exact binomial vs expected 0.06	One-tailed p < 0.001	**<0.001**
**Grouping**	**Category**	**N**	**Median survival (days) 95% CI**	**Statistical comparison**	**Test statistic**	**Test statistic**
Waterston comparison	B vs C	52		Breslow (generalized Wilcoxon)	χ2 = 1.99, df = 1	0.159
Pneumonia severity	None/mild		6.0 (3.73–8.27)			
Pneumonia severity	Moderate		5.0 (2.78–7.22)			
Pneumonia severity	Severe		3.0 (0.0–8.44)			
Pneumonia comparison	None/mild vs moderate vs severe	52		Breslow (generalized Wilcoxon)	χ2 = 1.174, df = 2	0.556
Birth-to-surgery timing	Operated within 1st week	10	7.0 (0.0–14.75)			
Birth-to-surgery timing	Operated in 2nd week	19	6.0 (4.78–7.22)			
Birth-to-surgery timing	Operated beyond 2nd week	23	4.0 (1.99–6.01)			
Birth-to-surgery comparison	1st week vs 2nd week vs > 2 weeks	52		Breslow (generalized Wilcoxon)	χ2 = 3.23, df = 2	0.199

Time-fixed binary logistic regression for 14-day postoperative mortality was conducted, including birth-to-surgery timing, pneumonia severity, and Waterston class as covariates. After adjustment, neonates operated within the first week had significantly lower odds of 14-day mortality than those operated beyond the second week (adjusted OR 0.34, 95% CI 0.12–0.97, p = 0.043). Infants operated in the second week had similar odds to those operated after the second week (adjusted OR 0.91, 95% CI 0.46–1.80, p = 0.781). Neither indicator of pneumonia severity reached statistical significance (moderate pneumonia aOR 1.90, 95% CI 0.65–5.54, p = 0.239; severe pneumonia aOR 1.66, 95% CI 0.49–5.59, p = 0.414). Waterston class C showed a non-significant trend toward lower odds compared with class B (aOR 0.45, 95% CI 0.19–1.05, p = 0.064) (Table 4 below). The logistic regression model demonstrated statistically significant overall fit (Omnibus χ² (5) = 12.613, p = 0.027), indicating that the included predictors collectively improved the prediction of 14-day postoperative mortality. The model showed moderate explanatory strength, with Cox & Snell R^2^ = 0.215 and Nagelkerke R^2^ = 0.358. The −2 Log Likelihood value of 35.303 further supports acceptable model fit for the sample size.

Survival time measured from date of surgery to death or censoring at 14 days; proportional hazards assumption was not satisfied for these comparisons so Breslow (generalized Wilcoxon) tests were used; exact one-sample binomial tests used Clopper–Pearson CIs; medians reported with 95% CIs.

## Discussion

This decade‐long review of 114 neonates with oesophageal atresia and tracheoesophageal fistula (OA/TOF) at a Tanzanian tertiary referral center reveals breakdowns across the care continuum, manifesting in high in‐hospital mortality that is typically seen in the sub-Saharan Africa [4,5,14,16]. We hereby apply the three delays model to view and interpret our results; [1] delays in recognition and misdiagnosis at peripheral facilities, [2] delayed transport and referral, and [3] delayed access to surgery after arrival due to ICU and perioperative constraints [21].

At the first and second delay, infants in our cohort waited a median of six days from birth to radiological confirmation of OA/TOF. During this period, repeated and unprotected feeding attempts almost certainly amplified the risk of aspiration, which is reflected in the high burden of pneumonia at diagnosis. The total number of confirmed OA/TOF cases over ten years (114 infants) was lower than epidemiological expectations for Tanzania, suggesting that a proportion of affected neonates were never recognized as surgical emergencies. As demonstrated in an Egyptian study [22] where 4% of neonates labelled as “sepsis” were ultimately found to have OA/TOF, misdiagnosis is a plausible contributor to undocumented first‑delay mortality in our setting. In our cohort, the clinical consequences of this first delay were evident: by the time OA/TOF was definitively diagnosed, only 35% of infants had mild pneumonia, while the majority had already progressed to moderate or severe disease [13]. These findings underscore how delays in early recognition and stabilization, and referral fuel the cascade of pulmonary injury that is so difficult to reverse in resource‐constrained neonatal units [6,15].

At the third delay, once determined to require an operative intervention at the tertiary facility, 54% of these infants succumbed before the operative intervention. Prolonged time to complete baseline investigations for associated anomalies— hereby inferred from the proportion that completed at least one preoperative investigation—and limited access to neonatal intensive care support may have exacerbated physiological deterioration, evidenced by refractory electrolyte imbalances and haematological derangements. The impact of associated malformations is likely underestimated, as evaluations for associated anomalies were performed selectively; consequently, the rates of determined associated anomalies were much lower than expected. Although birth weight and congenital heart disease have been widely recognized as important prognostic factors in well‐resourced settings, in our study they were not associated with preoperative mortality, possibly because their effects were muted here by the devastating impact of preoperative delays that drive pneumonia and sepsis [6,14,15]. Essentially, each additional day spent awaiting surgery contributed in eroding the fragile neonate’s physiological reserve, rendering conventional risk factors less predictive of outcome.

These third delays were shaped by several health‑system constraints. A major contributor was the limited availability of essential human resources. Although neonatologists, paediatric surgeons, and anaesthesiologists reviewed the neonates preoperatively, the availability of these speicalists are among the lowest in the region. During the study period there were no sub-specialized paediatric anaesthesiologist, less than 4 neonatologists, less than 4 paediatric surgeons— who still had to manage the burden of other childhood diseases during the study period; reflecting broader HRH shortages nationally, and regionally [13]. Infrastructure limitations further compounded these delays, including limited access to echocardiography and ultrasound slots, and the absence of fast‑track protocols specifically tailored for OA/TOF. Financial barriers also played a role, as some investigations required out‑of‑pocket payments. Additionally, time was required to correct deranged laboratory parameters and secure blood products before surgery. Collectively, these bottlenecks prolonged the preoperative phase; only after they were resolved could a theatre slot be sought.

Of those infants who underwent surgery, the mean interval from diagnosis to surgery was more than one week (8.5 days), substantially longer than the 24–48 hours typically recommended for stable OA/TOF cases in well-resourced settings [9,17]. Intraoperatively, we did not encounter Gross types B and D, which are reported in the literature to account for less than 1%. This may be due to their true rarity, or possibly they succumbed before operative intervention. Regarding Type E (the H-type), it is recognized that diagnosis is frequently missed during the neonatal period; therefore, these cases were not included in our study as they did not meet our radiological inclusion criteria [17]. Despite a primary anastomosis being achieved in 79% of cases, the 14-day postoperative mortality rate was strikingly high at 82.7%. Notably, a quarter of postoperative deaths occurred within the first 48 hours after surgery, and half by postoperative day six. Those operated within the first week had better survival because the physiological derangements from delays were least advanced. Earlier surgery not only avoids the development and progression of pneumonia to sepsis but also prevents catabolic deterioration.

The unexpectedly high postoperative mortality among Waterston class B neonates may be explained in the following ways firstly, progression of severe pneumonia and sepsis, driven by delayed diagnosis and aspiration [17], secondly, by the time these infants reached intervention, most had already experienced prolonged starvation, entering a catabolic state that impaired tissue healing and increased ventilatory requirements. Thirdly, possible underestimation of associated anomalies. These preoperative vulnerabilities were then compounded by limitations in postoperative intensive care capacity, including constrained monitoring, high nursing workload, and challenges in managing ventilated neonates, such as unrecognized endotracheal tube obstruction, malposition, or secretion accumulation [4,14,15]. Taken together these modifiable factors provide plausible explanation for the early postoperative deaths observed in Waterston class B neonates.

The better-than-expected survival for waterston class C further shows that it’s the modifiable risk factors that determine survival, no the nature of the anomaly primarily. We examined whether infants with obvious associated anomalies (Waterston class C) were referred earlier and therefore underwent earlier surgery; however, no such pattern was observed in our cohort.

Early surgical intervention was associated with better outcomes despite the contextual realities and serves a key area to build upon. Our findings align with multicountry LMIC data showing that delayed diagnosis and delayed surgery are major determinants of mortality [23,24]. Implementing locally feasible birth defects screening programs has the potential to improve early detection, which translates to better survival, especially for congenital anomalies that present as an emergency like OA/TOF. Although the logistic regression model demonstrated acceptable statistical fit, the low events-per-variable ratio limits the stability of coefficient estimates; therefore, findings should be interpreted as exploratory. The excluded 16 cases may have influenced mortality, but because this was the outcome data sensitivity analyses risked further bias.

These findings highlight a broader issue of justice and the right to surgical care for newborns with congenital anomalies. Because OA/TOF is uniformly fatal without operative repair, withholding surgery would remove any possibility of survival and effectively transfer the consequences of systemic resource limitations onto the infant. Offering surgery therefore remains ethically justified, even in a high-risk environment, as it preserves the child’s only chance of life. At the same time, the high mortality observed reflects structural inequities in neonatal intensive care, pediatric anesthesia, and perioperative capacity. By documenting where delays occur and how mortality accumulates, these data provide an evidence base for national advocacy, resource allocation, and international partnerships aimed at strengthening neonatal surgical systems. Addressing these structural injustices—not restricting access to life-saving surgery—is essential to advancing equity and child rights.

Improving survival will require coordinated feasible, locally sustainable national action that aims to: strengthen early detection at peripheral facilities through birth defects screening programs, establish time‑sensitive referral pathways, expand neonatal critical care capacity, and ensuring reliable access to essential diagnostics and perioperative support. These data provide an evidence base for advocacy, policy reform, and resource allocation aimed at reducing inequities in neonatal surgical care. Incremental but sustained improvements in health‑system capacity are essential if outcomes are to improve.

## Supporting information

S1 File STROBE Checklist. (DOCX)
