# Supplementary material for: Outcomes of infants undergoing surgery for oesophageal atresia and trachea-oesophageal fistula at a tertiary referral hospital in Tanzania
Source: PLOS Glob Public Health. 2026 Sep 10;6(9):e0005851. doi: 10.1371/journal.pgph.0005851 (PMC13561316; doi:10.1371/journal.pgph.0005851)
Supplement: Supplementary file 1 [file pgph.0005851.s001.docx]

**Outcomes of Infants Undergoing Surgery for Oesophageal Atresia and Trachea-Oesophageal Fistula at a Tertiary Referral Hospital in Tanzania**

**Strobe Checklist**

| Item No | Recommendation | Reported (Yes/No) | Manuscript location (section; page; pinpoint) |
| --- | --- | --- | --- |
| 1a | Indicate study design in title/abstract | Yes | Title and Abstract; page 1; Abstract Methodology: "Retrospective chart review…" |
| 1b | Provide informative summary in abstract | Yes | Abstract; page 1; Abstract Results paragraph (sample, preop & 14‑day postop mortality, OR 0.34) |
| 2 | Explain scientific background and rationale | Yes | Introduction; page 2; opening paragraphs describing burden and rationale |
| 3 | State objectives and prespecified hypotheses | Yes | End of Introduction; page 3; final paragraph states aims and outcomes of interest |
| 4 | Present key elements of study design early | Yes | Methodology opening; page 3; "A 10‑year retrospective chart review… Jan 2012–Dec 2022" |
| 5 | Describe setting, locations, and relevant dates | Yes | Methodology; page 3; Muhimbili National Hospital and study dates listed |
| 6a | Give eligibility criteria and selection methods | Yes | Methodology — inclusion/exclusion; page 3; radiologic confirmation and exclusion for missing outcome data |
| 6b | For matched studies give matching criteria | Not applicable | Methods; page 3; no matching performed |
| 7 | Define outcomes, exposures, predictors, confounders | Yes | Methodology — Variables & Definitions; pages 3–4; Waterston, pneumonia, timing, outcomes defined |
| 8 | Data sources and measurement methods for each variable | Yes | Methodology; pages 3–4; radiologic criteria and investigations described but measurement details limited |
| 9 | Describe efforts to address bias | Partially | Methods & Discussion; Methods lack specific bias‑reduction procedures; Limitations discuss exclusion and referral bias (Discussion pages 12–13) |
| 10 | Explain how study size was arrived at | Partially | Results; page 5; "130 charts screened, 114 included" but no sample size calculation |
| 11 | Explain handling of quantitative variables and groupings | Yes | Methodology; pages 3–4; birthweight, gestational age, timing categories and cutpoints specified |
| 12a | Describe statistical methods including confounding control | Yes | Methodology — Statistical analysis; pages 4–5; analyses time-fixed binary logistic regression |
| 12b | Methods for subgroup and interaction analyses | Yes | Methods — Survival & subgroup; pages 4–5; Breslow/log‑rank and subgroup comparisons described |
| 12c | Explain how missing data were addressed | Yes | Methods & Results; page 3 and page 5; listwise deletion stated and 16 charts excluded |
| 12d | Describe analytical methods accounting for loss to follow‑up | Yes | Methodology — Survival analysis; page 4; right censoring at 14 days described |
| 13a | Report numbers at each stage of study | Yes | Results; page 5; Figure 1 and first paragraph: 130 screened → 114 included → 52 operated |
| 13b | Give reasons for non‑participation at each stage | Yes | Results/Methods; pages 3–5; 16 excluded due to missing outcome data |
| 13c | Consider a flow diagram | Yes | Results; page 5; Figure 1 participant flow diagram provided |
| 14a | Give characteristics of study participants and exposures | Yes | Results — Baseline; pages 5–6; Table 1 shows demographics, pneumonia severity, investigations |
| 14b | Indicate number with missing data for each variable | Yes | Results; page 6; missingness described generally (e.g., ECHO 65%) variable‑by‑variable missingness table provided |
| 14c | Summarise follow‑up time (mean, total) | Yes | Methods & Results; pages 4 and 7; follow‑up defined (14‑day endpoint) and median survival reported |
| 15 | Report outcome events and summary measures | Yes | Results; pages 6–8; preop deaths (62/114), operated (52), 14‑day postop deaths (43/52) with KM median |
| 16a | Give unadjusted and adjusted estimates with precision | Yes | Results; pages 7–8; unadjusted proportions and adjusted OR reported |
| 16b | Report category boundaries when continuous variables categorized | Yes | Methods; pages 3–4; explicit cutpoints for birthweight, gestational age, timing categories |
| 16c | If relevant, translate relative risk into absolute risk | Yes | Results; pages 6–8; absolute risks provided (e.g., 54% preop mortality; 82.7% 14‑day postop) |
| 17 | Report other analyses (subgroups, sensitivity) | Partially | Results; pages 7–8; subgroup survival analyses provided but no sensitivity analyses for excluded charts |
| 18 | Summarise key results with reference to objectives | Yes | Discussion opening; page 11; restates aims and summarizes findings |
| 19 | Discuss limitations including potential bias and imprecision | Yes | Discussion — Limitations; pages 12–13; retrospective, single‑centre, exclusion bias and unmeasured prereferral deaths |
| 20 | Provide cautious overall interpretation considering limitations | Yes | Discussion; pages 11–14; contextualised interpretation and policy implications |
| 21 | Discuss generalisability (external validity) | Yes | Discussion; page 13; paragraph addresses applicability to national/LMIC contexts |
| 22 | Give funding sources and role of funders | Yes | Declarations; page 14; funding from RCSI/COSECSA 2022 Collaboration Programme and Irish Aid described |
| 23 | Report ethical approval and consent procedures | Yes | Methodology / Ethics; page 4; MUHAS-REC-01-2023-1513 and MNH/TRCU/IRB/Perm/2023/256 specified and waiver of consent stated |
